# Heated rivalries: Phenological variation modifies competition for pollinators among arctic plants

**DOI:** 10.1111/gcb.15303

**Published:** 2020-09-11

**Authors:** Mikko Tiusanen, Tuomas Kankaanpää, Niels M. Schmidt, Tomas Roslin

**Affiliations:** ^1^ Spatial Foodweb Ecology Group Department of Agricultural Sciences University of Helsinki Helsinki Finland; ^2^ Department of Bioscience Aarhus University Roskilde Denmark; ^3^ Arctic Research Centre Aarhus University Aarhus C Denmark; ^4^ Department of Ecology Swedish University of Agricultural Sciences Uppsala Sweden

**Keywords:** arctic ecology, climate change effects, competition for pollination, flowering phenology, indirect competition, phenology shift, pollination

## Abstract

When plant species compete for pollinators, climate warming may cause directional change in flowering overlap, thereby shifting the strength of pollinator‐mediated plant–plant interactions. Such shifts are likely accentuated in the rapidly warming Arctic. Targeting a plant community in Northeast Greenland, we asked (a) whether the relative phenology of plants is shifting with spatial variation in temperature, (b) whether local plants compete for pollination, and (c) whether shifts in climatic conditions are likely to affect this competition. We first searched for climatic imprints on relative species phenology along an elevational gradient. We then tested for signs of competition with increasing flower densities: reduced pollinator visits, reduced representation of plant species in pollen loads, and reduced seed production. Finally, we evaluated how climate change may affect this competition. Compared to a dominant species, *Dryas integrifolia *×* octopetala*, the relative timing of other species shifted along the environmental gradient, with *Silene acaulis* and *Papaver radicatum* flowering earlier toward higher elevation. This shift resulted in larger niche overlap, allowing for an increased potential for competition for pollination. Meanwhile, *Dryas* emerged as a superior competitor by attracting 97.2% of flower visits. Higher *Dryas* density resulted in reduced insect visits and less pollen of *S. acaulis* being carried by pollinators, causing reduced seed set by *S. acaulis*. Our results show that current variation in climate shifts the timing and flowering overlap between dominant and less‐competitive plant species. With climate warming, such shifts in phenology within trophic levels may ultimately affect interactions between them, changing the strength of competition among plants.

## Graphical abstract

For flowering species, it is not only important to be in temporal synchrony with the potential pollinators, but to also avoid the competition for pollination by other more attractive species. In the Arctic, changing climatic conditions change the relative flowering phenology of species, thus changing the overlap of their flowering and affecting the strength of the competition for pollination. Increasing overlap with dominant flowering species, *Dryas integrifolia* × *octopetala*, reduces flower visits, pollen transport, and seed set of *Silene acaulis*.

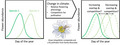

## 1 INTRODUCTION

Flowering plants and their pollinators form complex networks of interactions (Bascompte & Jordano, 2007; Jordano, 1987). While such networks are typically framed in terms of mutualistic interactions between the two trophic levels, within‐guild interactions offer an important added dimension (Blüthgen & Klein, 2011; Carvalheiro et al., 2014). Most of these within‐guild interactions have been described as competitive in character (with plants species e.g., competing for pollinators; Bartomeus, Vilà, & Santamaría, 2008; Brown, Mitchell, & Graham, 2002; Goodell & Parker, 2017; Mitchell, Flanagan, Brown, Waser, & Karron, 2009), but some are clearly facilitative (with e.g., one plant species attracting pollinators, which will then visit other species locally, Ghazoul, 2006; Losapio et al., 2019; Waser & Real, 1979). However, the individual‐, population‐, and community‐level consequences of these intraguild interactions have remained less explored (but see e.g., Bell, Karron, & Mitchell, 2005; Campbell & Motten, 1985).

Generally, wild plants share their flowering period with many other temporally overlapping plant species (e.g., CaraDonna et al., 2017; Mosquin, 1971). Thus, competition by simultaneously flowering species could hamper the reproduction of a focal species through increased competition for pollinators. For many insect‐pollinated plants, it is crucial to gain access to pollination services provided by local pollinators. As a consequence, Mosquin (1971) hypothesized that species have evolved differing flowering times to increase their fitness by reducing interspecific competition for pollinators. Especially more abundant and attractive, dominant plant species are expected to reduce the fitness of less attractive species when flowering at the same time (Carvalheiro et al., 2014; Goodell & Parker, 2017; Montero‐Castaño, Ortiz‐Sánchez, & Vilà, 2016). However, neutral and facilitative effects have also been observed (Gilpin, Denham, & Ayre, 2019; Holzschuh, Dormann, Tscharntke, & Steffan‐Dewenter, 2011; Lázaro, Lundgren, & Totland, 2009).

At present, climate change is shifting not only the phenology of individual species, but also the relative phenology of interacting species (CaraDonna, Iler, & Inouye, 2014; Høye, Post, Schmidt, Trøjelsgaard, & Forchhammer, 2013; Kudo & Cooper, 2019; Rafferty, Diez, & Bertelsen, 2020). Thus, individual species have to simultaneously adjust both to new abiotic conditions and to changes in the species with which they interact (Burkle & Alarcón, 2011; Kaiser‐Bunbury, Muff, Memmott, Müller, & Caflisch, 2010; Saavedra, Rohr, Olesen, & Bascompte, 2016). In terms of pollination, progressing climate change may cause the timing of flowering in competing plant species to slide toward larger or smaller overlap (e.g., CaraDonna et al., 2014; Forrest, Inouye, & Thomson, 2010; Schmidt et al., 2016), as a likely consequence of interaction partners responding to different environmental cues. Consequently, shifts in the relative phenology of flowering may change the strength of intraguild competition and fitness of individual species. Yet, such effects remain poorly studied (but see Giejsztowt, Classen, & Deslippe, 2019; Kehrberger & Holzschuh, 2019). These changes in relative phenology are expected to come with more drastic consequences when the diversity is low (offering less potential for interaction rewiring; Benadi, Hovestadt, Poethke, & Blüthgen, 2014) or the flowering is highly seasonal (resulting in more dramatic mismatches in systems without "background noise" of flowers and pollinators; Pelayo, Soriano, Márquez, & Navarro, 2019).

The Arctic is a region where the above effects are likely accentuated (Høye, Post, Meltofte, Schmidt, & Forchhammer, 2007; Iler, Høye, Inouye, & Schmidt, 2013). Here, the growing season is short, and all flowering thus confined to a relatively short time window. Moreover, the growing season has shrunk even shorter with progressing climate warming (Høye et al., 2013; Prevéy et al., 2019). Since climate change is particularly rapid in the Arctic (due to so called arctic amplification; Kattsov et al., 2005), we may expect to see climate‐induced intensification in plant–plant competition for pollinators (Cirtwill, Roslin, Rasmussen, Olesen, & Stouffer, 2018; Post et al., 2009). Mimicking trends seen elsewhere (Cardoso et al., 2020; Powney et al., 2019), several arctic pollinators have also suffered recent population decline (Gillespie et al., 2019; Loboda, Savage, Buddle, Schmidt, & Høye, 2017). Hence, at high latitudes we may expect particularly strong competition for pollinators, and particularly accentuated shifts in interspecific competition for pollination with climate change.

In this study, we focus on a pollination community of the High Arctic. Focusing on six quantitatively dominant plant species and their associated pollinators in the Zackenberg valley of Northeast Greenland, we ask (a) whether the relative phenology of local plants is shifting along temperature gradients, (b) whether local plants compete for pollination, and (c) whether altering climatic conditions are likely to affect this competition in space and time. To this aim, we first examine the climatic imprints on relative phenology of flowering species along an elevational gradient (representing current climatic variability). We then search for signs of intensified competition with increasing flower densities, expressed as reduced visits per flower, reduced representation of plant species in pollen loads, and a reduced proportion of inflorescences producing seeds. The latter analysis was specifically focused on two species identified through our data on pollinator visits: *Dryas integrifolia *×* octopetala* Rosaceae as a plant attracting a particularly high proportion of insect visits, and *Silene acaulis* Caryophyllaceae is a gynodioecious plant with some proportion of individuals being female only. Such individuals will exhibit a particularly high demand for pollination, since self‐pollination is—per definition—excluded. After testing for both phenological shifts in flowering time along environmental gradient and competition for pollination among the flowering species, we infer how the changing climate is likely to translate into shifts in the competitive landscape, and ultimately fitness, of local species.

## 2 MATERIALS AND METHODS

To examine spatiotemporal patterns of flowering abundance, phenology, and interspecific overlap, we targeted sites along an elevational gradient in a high arctic system: the Zackenberg valley in NE Greenland (74°28′N, 20°34′W). At each site, we monitored the abundance of flowers at weekly intervals. To relate these flower abundances to interspecific competition for pollinators, we monitored multiple stages of the pollination process (Ne'eman, Jürgens, Newstrom‐Lloyd, Potts, & Dafni, 2010) among the dominant flowering plant species: insect visitation rates (i.e., pollinator visits per flower and time unit), pollen transport (i.e., the representation of the species in pollen loads carried by pollinating flies), and seed set success (i.e., the proportion of inflorescences setting seed). We then searched for climatic imprints on relative species phenology along the elevational gradient (representing current climatic variation, with elevation as a space‐for‐time surrogate for climate change; e.g., Benadi et al., 2014; Elmendorf et al., 2015; Hoiss, Krauss, & Steffan‐Dewenter, 2015; Kearns, 1992; Körner, 2007).

### 2.1 Study species

To characterize variation in the phenology of flowering and competition for pollinators among plant species, we counted inflorescences of all flowering species. Six species emerged as quantitatively dominant in the local flora: *Cassiope tetragona* Ericaceae, *D. integrifolia *×* octopetala* Rosaceae, *Papaver radicatum* Papaveraceae, *Salix arctica* Salicaceae, *Saxifraga oppositifolia* Saxifragaceae, or *S. acaulis* Caryophyllaceae. These species are all abundant and widely distributed across the Arctic (Walker et al., 2005).

### 2.2 Study sites

We recorded the flowering of plants and flower visitor abundance at 24 study sites (50 m × 50 m each) from late June to early August in 2016. In order to track the effects of the local variation in climate along the elevation gradient on plant–pollinator interactions in a space‐for‐time experiment, we chose eight study sites in each of three zones along an elevation gradient (0–60, 60–240, and 240–480 m above sea level, 3 × 8 = 24 sites total; see Figure S1). To characterize temperature conditions across the gradient, we drew on records from another study (Kankaanpää, 2020), with data presented in Figure S2. To minimize the generally large effects of changing plant community on pollination along the elevation gradient (Simanonok & Burkle, 2014), each of the sites represented the same vegetation type, *Dryas* heath. This vegetation type is abundant and widespread at all elevations considered (Bay, 1998). To avoid effects caused by spatial variation in the timing of snow‐melt (Kankaanpää et al., 2018; Kudo & Hirao, 2006), the study sites were established in areas at the same phenophase, that is, when the first flowers opened. The study sites were separated by distances of at least 250 m, a scale over which we assumed few arctic insects to move during their daily foraging. Thus, the sites were considered at least semi‐independent in terms of their insect populations. Within each of the 24 study sites, we marked 10 study plots (circular, radius 50 cm) with small flags to locate them later.

### 2.3 Phenological variation in flower densities and insect visitation

Once a week, we recorded the local, instantaneous density of all flowering plant species at two spatial scales: at the level of the study site and at the level of the study plot. The rate with which different insects visited flowers as a function of contemporary flower densities was scored twice a week by visual observation. During each visit, we walked up to a distance of 2 m from the plot and recorded all visitors on the flowers (thus scoring a snapshot of arthropod abundances present on the flowers upon the observer's arrival). This was, on average, achieved in just some minute per site. The radius of the area for visitor inspection equaled two visual fields of the binoculars used (Ibis, model 10 × 42; Kite), that is 50 cm, with the mark flag held at the center of the circle. Within this area, we recorded all flower visitors at the family level. Flower visitation observations were done mainly between 10:00 and 18:00 and only if the weather conditions were suitable for flower visitors (no rain or heavy wind).

### 2.4 Pollen transport by flies

To establish the impacts of flower densities on pollen transport (i.e., the representation of pollen from focal flower species in pollen carried by insects), we focused on pollen loads on flies in family Muscidae. This taxon was chosen for being the presumptively most important pollinators in the area (and many other arctic and alpine areas as well; Kearns, 1992; Kevan, 1972; Pont, 1993; Tiusanen, Hebert, Schmidt, & Roslin, 2016), and the numerically dominant fly taxon of the High Arctic (Böcher, Kristensen, Pape, & Vilhelmsen, 2015; Loboda et al., 2017).

With the aim of examining how the pollen loads reflected plant species‐specific flower densities and relative flowering phenology, we captured 10 fly individuals at each of the study sites every week. The flies were individually caught with an insect net while they were basking on vegetation or soil on the study sites. To avoid interfering with local flower visitation patterns, and to prevent secondary contamination by pollen during handling, we explicitly avoided catching insects sitting on flowers. All flies caught were stored individually in ethanol‐filled tubes.

To remove the pollen from the flies, we then vortexed the tubes (max rpm for 10 s, Vortex‐Genie 2, Scientific Industries, Inc.). To concentrate the pollen in the bottom of the tube, the fly was removed, and the pollen suspension centrifuged at 3,000 *g* for 3 min (Sigma Laboratory centrifuges, model 4‐15C). In order to count and identify the pollen, we evaporated the ethanol and cleaned the dry tube from pollen with the aid of agarose gel (15 ml glycerin, 25 ml water, 0.5 g agar, red food dye, Dr. Oetker), which was then poured onto a microscopy slide. The pollen samples were identified and counted with a microscope (CX41, Olympus). Due to the difficulty of identifying the muscid flies in the field, we stayed with family‐level identification.

### 2.5 Proportion of inflorescences setting seed

To estimate the impact of competition on the seed set of plants, we chose two abundant and widely distributed species: *D. integrifolia *×* octopetala*, henceforth *Dryas* for brevity, and *S. acaulis*. Of these, *Dryas* was selected as a particularly dominant species in the plant community with a need for pollinators for optimal seed set (Tiusanen et al., 2016) and *S. acaulis* is a plant with a particularly high demand for pollen transport services: *S. acaulis* is gynodioecious, with some plants being hermaphrodites and others female only (Kevan, 1972; Shykoff, 1992). Thus, in *S. acaulis*, we used female‐only plants with an obligate need for pollinators to score whether they got successfully pollinated or not. For this purpose, we targeted a subset of study sites with sufficient abundances (>500 flowers per site) of these flowering species (14 and 10 sites for *Dryas* and *S. acaulis*, respectively). To keep track of spatiotemporal variation in the seed production, we marked 10 flowers per species (female‐only individuals of *S. acaulis*) at each targeted site each week. All the marked flowers were recently opened (no more than 24 hr old). During the first visit to the study sites, we also found *Dryas* flowers, which were already senescent (at 14 of the sites) and withered (at 13 of the sites). At each of these sites, we marked 10 old and 10 withered flowers (in addition to freshly opened flowers). Reflecting the average flowering time of *Dryas* (M. Tiusanen, personal observation, June–July, 2016), we subsequently treated them as if they had first opened 2 or 6 days, respectively, before our visit.

To resolve the effect of pollinator availability on the seed set of *Dryas*, and to probe for differences in seed set over time, we excluded pollinators from accessing some of the flowers. We did so by covering 10 unopened buds with plastic cups (Iisi, 0.25 L, Nupik International). To minimize the effects of the treatment on temperature and moisture, the bottom of which had been replaced with a mesh (mesh size 0.3 mm × 0.3 mm, Yleistylli, pehmeä, Eurokangas). Wherever possible, we chose flowers on the same tussocks as the flowers monitored for seed set in the presence of pollinators. For *S. acaulis*, we relied on the self‐sterility of the female‐only individuals targeted (see above). Seed set by such individuals reflects successful pollen transport from another individual. Given the spatial distribution of plants in the study system, it will almost invariably require an insect vector.

At the end of the season, we investigated seed set success of *Dryas* and *S. acaulis* (i.e., whether the flower had produced seeds or not) of the marked flowers, and—for *Dryas*—of the flowers from which pollinators had been excluded.

### 2.6 Statistical methods

#### 2.6.1 Relative phenology of flowering species

To describe phenological patterns in the flowering of different plant species, we calculated the mean date of flowering for each of the flowering species at each of the study sites as the mean occurrence of open flowers $\left(\frac{\sum_{k=1}^{n}{\text{DOY}}_{k}*\text{number}\;\text{of}\;{\text{flowers}}_{k}}{\sum_{k=1}^{n}\text{number}\;\text{of}\;{\text{flowers}}_{k}}\right)$, expressed as day of year, DOY. The overall DOY of the mean flowering of a plant species was calculated as the average of all the site‐specific values.

To examine how the relative timing of flowering in other plant species along the elevational gradient, we fitted generalized linear models (GLMs) in *R* (The R Core Team, 2016) to phenological data. Since *Dryas* emerged as the most attractive species (see Section 3) we used the difference of the date of mean flowering between *Dryas* and the focal species (*C. tetragona*, *P. radicatum*, *S. arctica*, *S. oppositifolia*, or *S. acaulis*) as the response variable and elevation as an explanatory variable. Sites with observations of a species flowering only on 1 day were excluded from the species‐specific analyses, as offering records with disproportionately low precision.

Notably, the key interest here relates to the interaction “plant species” × “overall annual phenology.” A significant interaction will reveal differential responses in different species, and the slope estimates will indicate the extent to which flowering in different species gets compressed or spread aside by variation in the relative earliness of the year. In other words, the interaction will quantify the impact of climate on the overlap in timing among competing species. To account for environmental variation causing site‐to‐site differences in flowering time, we used study plot as a random effect. The model was fitted with package *lme4* (Bates et al., 2017) in *R* (The R Core Team, 2016).

To further examine the potential for competition between the plant species, we characterized the site‐specific temporal niche overlap in flowering between *Dryas* and other species with Schoener's index (SI, Schoener, 1970). $\text{SI}=1‐\frac{1}{2}\sum_{i}\left|p_{\mathrm{xi}}‐p_{\mathrm{yi}}\right|$, where *p_xi_* and *p_yi_* are the normalized flower abundances of species *x* and *y* on day *i*, respectively (SI gets values between 0 and 1, with higher values indicating larger overlap). To test for a change in niche overlap with *Dryas* along the elevation gradient, we modeled the SI between *Dryas* and the focal taxon (*C. tetragona*, *P. radicatum*, *S. arctica*, *S. oppositifolia*, *S. acaulis* or the average of SI of all species) as a function of elevation. These GLMs were fitted in R (The R Core Team, 2016). Sites with observations of a species flowering only on one day were excluded from the species‐specific analyses, as offering records with disproportionately low precision.

#### 2.6.2 Visitation rate as a function of flower densities

If with more flowers of the most attractive species (*Dryas*), there would be no signs of less visitors per flower, then one might hardly argue that pollinators in this system are in limited supply. Therefore, we tested whether intensified competition with increasing flower densities was evident as reduced visits per flower, focusing on the most highly visited plant, *Dryas*. To account for a high number of zero observations, we fitted a hurdle model to the counts of flower visitors as a function of *Dryas* flower abundance on the respective study plot. The number of *Dryas* flowers was used as an offset in the model. For the count model, we assumed a Poisson distribution and a log‐link function, and for the zero model we used a binomial distribution with a logit‐link function. The model was fitted with package *pscl* (Jackman et al., 2017) in *R* (The R Core Team, 2016).

#### 2.6.3 Pollen transport by flies

**FIGURE 1:**
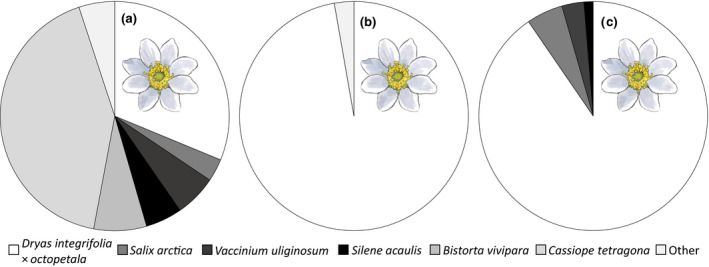
Relative dominance of plant species among (a) accessible flowers, (b) number of visitations by insects, and (c) pollen transported by muscid flies. Shown in (a) is the fraction of flowers of different plant species across the study sites (total *n* = 1,313,960 inflorescences). Shown in (b) is the fraction of insect visits to different plant species (total *n* = 2,287 flower visitors). Shown in (c) is the fraction of pollen grains of different plant species observed across 797 muscid fly individuals (total *n* = 206,917 pollen grains). For panel (a) resolved by elevational zones, see Figure S3

Since *Dryas* attracted the vast majority of flower visits (Figure 1), we chose to focus on the effect of *Dryas* flower densities on the transport of conspecific and heterospecific pollen. These analyses were focused on *S. acaulis*, as the species with the highest level of co‐occurrence of pollen with *Dryas* in space (across sites) and time. For other plant species, the overall incidence of pollen transport by muscids proved too low and/or spatiotemporal co‐occurrence with *Dryas* too scarce to allow meaningful analyses (see Figure 1 for a visual representation of the dominance of *Dryas*). To test whether high densities of *Dryas* flowers affect pollen transport by muscid flies, we modeled fly transport of *Dryas* and *S. acaulis* pollen, respectively, by generalized linear mixed effect models (GLMMs) fitted separately to data on each plant species. The presence or absence (1/0) of *Dryas* pollen on individual flies (one observation per fly) was modeled as a function of the day‐ and site‐specific abundance of *Dryas* flowers and *S. acaulis* flowers on the study sites (treated as continuous, fixed effects; the model was fitted for the presence or absence of the pollen instead of actual pollen counts to avoid overdispersion). The flower abundances on specific DOYs were acquired from field observations (see above) or—where the abundance was not recorded on the specific date—by linearly interpolating missing values between observed ones.

To capture competition of a form where increasing abundances of flowers of a species results in a decreasing probability of flies carrying heterospecific pollen (as a sign of competition for pollinators), we included the interaction between *Dryas* and *S. acaulis* flower abundances. Because the effect of the local flower abundance on pollen loads carried by flower visitors can be expected to saturate (a flower visitor cannot carry an unlimited amount of pollen or visit all the flowers), flower abundances were log_10_(*n* + 1)‐transformed. To account for site‐to‐site differences in pollen loads, we included study site as a random effect. Since the dependent variable was a proportion of events, we assumed a logit‐link function and binomially distributed errors. The presence or absence (1/0) of *S. acaulis* pollen was then modeled by an equivalent GLMM. The models were fitted package *lme4* (Bates et al., 2017) in R (The R Core Team, 2016). Because monitoring of study sites V, W, and X (see Figure S1) did not start from the beginning of the season, they were excluded from the analyses.

#### 2.6.4 Seed set by inflorescences

To resolve temporal patterns in seed set, we used GLMMs of the fraction of *Dryas* and *S. acaulis* inflorescences, respectively, producing seeds as a function of DOY. Because *Dryas* attracts a majority of flower visits, high densities of *Dryas* could potentially reduce the seed set of other flowers. Therefore, we also modeled the seed sets of *Dryas* and *S. acaulis* as a function of the local abundance of open *Dryas* flowers on the study site. The *Dryas* abundances on specific DOYs were acquired from field observations or—where the abundance was not recorded on the specific date—by linearly interpolating missing values between observed ones. To account for environmental variation causing site‐to‐site differences in average seed set, we included study site as a random effect. To clarify the role of pollinators on the seed set of *Dryas* and to test for experimental artifacts induced by seasonal changes in seed set unrelated to pollinators, we modeled the fraction of inflorescences producing seeds as a function of the exclusion treatment, DOY, and their interaction. To account for environmental variation causing site‐to‐site differences in average seed set, we included study site as a random effect. The models were fitted package *lme4* (Bates et al., 2017) in R (The R Core Team, 2016). Because the dependent variable was a proportion of events, we assumed a logit‐link function and binomially distributed errors.

## 3 RESULTS

We counted a total of 1,310,526 flowers (or inflorescences) within our study sites. The most abundant flowering plants were *C. tetragona* Ericaceae, *D. integrifolia *×* octopetala* Rosaceae, and *Bistorta vivipara* Polygonaceae, accounting for 42.0%, 31.2%, and 7.4% of all flowers, respectively (Figure 1a).

The contemporary flowering phenology varied substantially among plant species. *S. arctica* flowered the earliest, with a mean flowering date of DOY 174, whereas mean flowering of *Dryas* occurred at DOY 181 and mean flowering of *S. acaulis* at DOY 184. *Bistorta vivipara* was the most abundant flower in the late season, with a mean flowering DOY of 201.

### 3.1 Elevation affects relative flowering phenology and niche overlap

**FIGURE 2:**
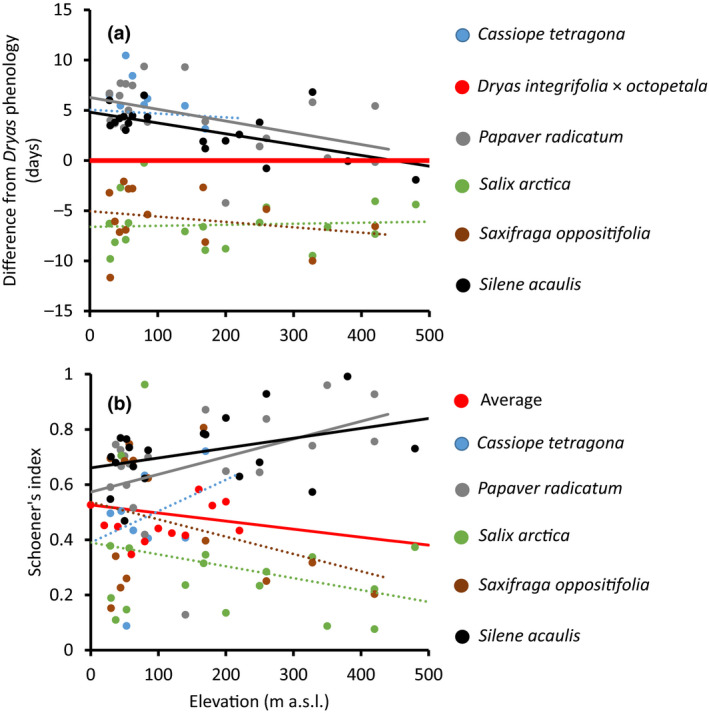
Phenological overlap between *Dryas integrifolia *×* octopetala* and other flowering species as a function of elevation. In (a), we show the difference in the day of year (DOY) of mean flowering of the species compared to *Dryas* (equaling *Y* = 0; shown as thick red line for comparison), with the lines showing the fitted linear models described in the text. The DOY of mean flowering is represented using *Dryas* as the reference group because of its dominant role in the pollination network. Slopes significantly different from that of *Dryas* are indicated with solid lines. In (b), we show the value of Schoener's index of temporal niche overlap between *Dryas* and other species (high values indicate larger niche overlap), with the lines showing the fitted linear models described in the text. Significant slopes are indicated with solid lines

Importantly, the relative timing of *Dryas* and *S. acaulis* shifted along the spatial gradient, with *S. acaulis* flowering later in relation to *Dryas* toward lower elevation (Figure 2a). Along the gradient in elevation, the flowering of *S. acaulis* delayed compared to flowering of *Dryas* by 1.1 days per 100 m decrease in elevation (*t*_19_ = 3.2, *p* = .005). Meanwhile, compared to *Dryas*, the relative phenology of *P. radicatum* was increasingly delayed toward lower elevation (*t*_20_ = 2.2, *p* = .04). The phenology of *C. tetragona*, *S. arctica*, or *S. oppositifolia* showed no patterns in relation to the phenology of *Dryas* along the elevation gradient.

As a consequence of shifts in phenology, the niche overlap with *Dryas* showed variation along the elevation gradient (Figure 2b). On average, niche overlap decreased with increasing elevation (*t*_24_ = −2.8, *p* = .01). However, *P. radicatum* (*t*_20_ = 2.2, *p* = .05) showed an increase and *S. acaulis* a marginal increase (*t*_19_ = 1.7, *p* = .11) in niche overlap with *Dryas* with increasing elevation. The niche overlap (SI) of *Dryas* and *C. tetragona*, *S. arctica*, or *S. oppositifolia* showed no detectable patterns along the elevation gradient.

### 3.2 Arctic plants compete for pollination

**FIGURE 3:**
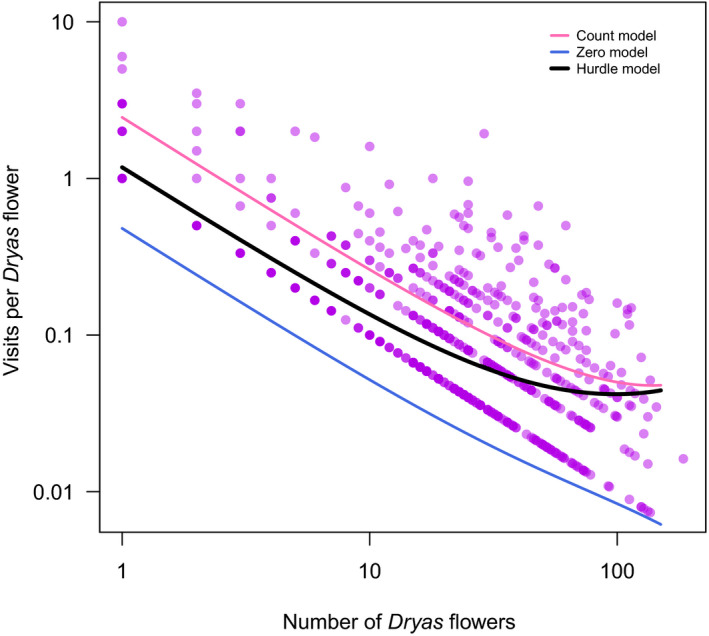
Competition for flower visitors. Shown is the number of insect visits per *Dryas integrifolia *×* octopetala* flower as a function of the number of *Dryas* flowers per study plot. Each data point represents a date‐specific observation at a particular study plot (*n* = 1,151). Pink, blue, and black line represent estimates from a hurdle model fitted to the data, with the blue line representing the zero model, the red line the count model, and the black curve the hurdle model combining the effects of these two. Note the logarithmic scale on the *x*‐axis and the *y*‐axis

Of a total of 2,287 observed flower visitations, a highly disproportionate fraction (97.2%, i.e., 2,224 visits) occurred on a single species, *Dryas* (Figure 1b), despite the fact that within our study plots (surveyed within the study sites), *Dryas* accounted for only 56.2% of the flowers. Thus, *Dryas* flowers were massively overrepresented among the visits compared to the other relatively abundant species, including *Silene*. Overall, high number of flowers of the most attractive species (*Dryas*) caused potential shortage of pollination, as the number of flower visits on individual plants decreased as a function of the number of flowers (*z* = −1,500, *p* < 2*10^–16^; Figure 3), with visitation rates on *Dryas* flowers being highest during the early season, before the flowering peak.

**FIGURE 4:**
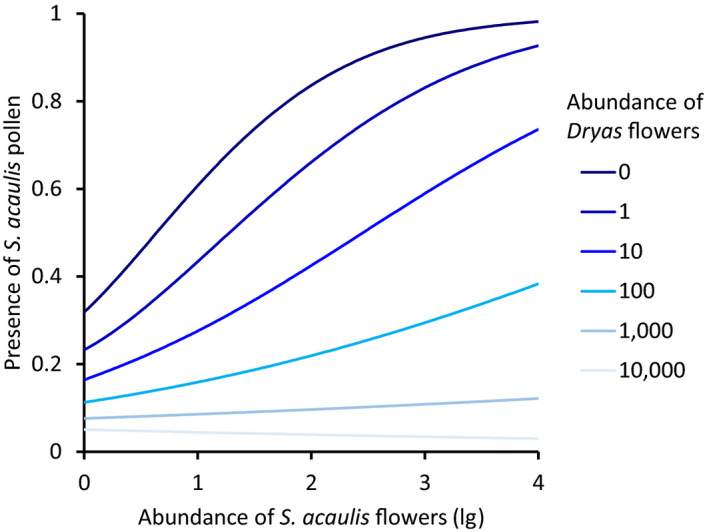
Competition for pollen transport. Shown is the presence of *Silene acaulis* pollen on muscid flies as a function of *S. acaulis* and *Dryas integrifolia *×* octopetala* flower abundances. The *y*‐axis shows the probability with which *S. acaulis* pollen was found on individual muscid flies, while the *x*‐axis shows log10(*n* + 1)‐transformed *S. acaulis* flower abundance. Lines of different colors represent different abundances of *Dryas* flowers, and reveal the strong interaction: at high densities of *Dryas*, the flower abundance of *S. acaulis* will essentially have little effect on the probability with which a fly carries *S. acaulis* pollen. The graph shows fitted probabilities from the GLMM described in the text

Dominance of *Dryas* was also evident in pollen loads carried by flies in family Muscidae. Overall, we identified and counted 206,917 pollen grains carried by 763 of 797 the captured muscid fly individuals. Of this total, 90.4% of grains was morphologically identified as *Dryas* (Figure 1c). The effects of abundance of plant species on the pollen transport of each other were highly asymmetrical: The proportion of muscid flies carrying pollen of *Dryas* increased with an increasing abundance of *Dryas* flowers in the surrounding study site (odds ratio, OR: 1.66; 95% confidence interval: 1.40–1.98). However, the abundance of *S. acaulis* flowers did not have any detectable effect of the probability of pollinators to carry *Dryas* pollen (OR: 0.65; 0.32–1.32), and did not modify the effect of *Dryas* abundance on pollen transport of *Dryas* (OR: 1.03; 1.01–1.04). Likewise, the probability of a muscid fly carrying pollen of *S. acaulis* increased with an increasing abundance of *S. acaulis* flowers (OR: 3.31; 2.52–4.35; Figure 4). However, it decreased with increasing abundance of *Dryas* flowers (OR: 0.64; 0.57–0.85; Figure 4), and even the positive relationship with *S. acaulis* own flower density disappeared when surrounded by a high abundance of *Dryas* flowers.

**FIGURE 5:**
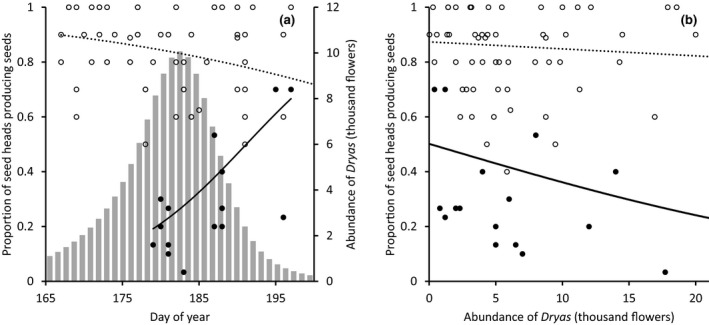
Seed set success of *Dryas integrifolia *×* octopetala* and *Silene acaulis* (a) as a function of day of flowering and (b) as a function of abundance of *Dryas* during flowering. The solid line and the dashed line represent seed set success of *S. acaulis* and *Dryas*, respectively, as derived from the generalized linear mixed effect model described in Section 2. Observed seed set success (i.e., proportion of inflorescences producing seed) of *S. acaulis* and *Dryas* are shown by black and open circles, respectively. In (a), the gray bars represent the abundance of *Dryas* as a function of day of year, with the scale on the on the right‐hand abscissa

As a potential result of competition for pollinators, the proportion of inflorescences producing seeds was affected both by pollinator access and by flower densities during the flowering. At the end of the season, we inspected a total of 921 and 627 flower heads of *Dryas and S. acaulis*, respectively. Six hundred and ninety‐three *Dryas* and 223 *S. acaulis* seed heads had successfully generated seeds (75.2% and 35.7%, respectively). Seed set of *Dryas* decreased as a function of the day of flowering (*z* = −2.78, *p* < .01; Figure 5a), whereas that of *S. acaulis* increased (*z* = 8.1, *p* < .001; Figure 5a). In addition, the seed set of *S. acaulis* decreased with increasing abundance of *Dryas* flowers (*z* = −8.3, *p* < .001; Figure 5b). Meanwhile, the seed set of *Dryas* was not affected by the abundance of *Dryas* flowers (*z* = 1.08, *p* = .28; Figure 5b).

**FIGURE 6:**
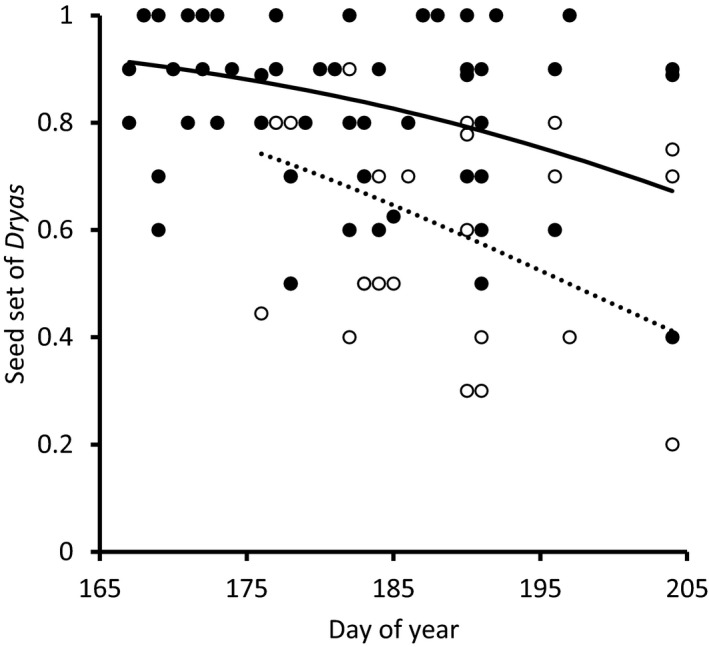
Seed set success of *Dryas integrifolia *×* octopetala* as a function of day of flowering. The solid line and the dashed line represent seed set success of flowers without and with cups used to exclude pollinators from accessing the flowers, as derived from the generalized linear mixed effect model described in Section 2. Observed seed set success of *Dryas* flowers with and without access to pollinators are shown by black and open circles, respectively

Among the 328 *Dryas* inflorescences from which pollinators were excluded, seed set was reduced as compared to flowers to which pollinators had access (*z* = −2.05, *p* = .04, Figure 6). While the seed set of *Dryas* was lower toward the end of the season (OR: 0.96; 0.89–0.98), there was no significant interaction between the DOY and the exclusion treatment (OR: 0.95; 0.97–1.02). While pollinator exclusion consistently decreased seed set across the season, there was also an effect of DOY, as the proportion of seed set attributable to presence of pollinators increases toward the end of the growing season (difference of no‐exclusion and exclusion, e.g., DOY 167:9.4%, DOY 204 26.1%, Figure 6).

## 4 DISCUSSION

If different plants rely on different environmental cues to initiate flowering, then progressing climate change may cause the timing of interacting individual species to slide toward more or less overlap (CaraDonna et al., 2014; Schmidt et al., 2016). Where plants compete for pollinators, such shifts in relative timing may either increase or decrease the strength of competition. In this study, we found that pollinators in the target area is in short supply, as evidenced by decreasing visitation rates with higher flower densities (Figure 3) and by decreasing seed set with the exclusion of pollinators (Figure 6). As a result, plants compete for pollinators—which is evidenced in decreasing visitation rates with a higher abundance of the more‐attractive Dryas (Figures 1 and 3), in a declining representation of the less‐competitive *S. acaulis* in pollen loads (Figure 4) and in patterns of seed set (Figure 5). Finally, this competition is likely affected by the shifting climate along the elevation gradient which modifies the intensity of the competition through changes in phenological overlap between the flowering species (Figure 2). Below, we will examine each of these findings in turn.

### 4.1 Elevation affects relative flowering phenology and niche overlap

During the arctic summer, flowering advances quickly, with the peak flowering period at Zackenberg lasting less than 3 weeks. Within such a short summer, it is crucial for plants to reproduce efficiently. Thus, plants should time their flowering to maximize pollination while avoiding competition for the pollinators. Different plants species indeed differ in their phenological timing, but the extent of overlap differs in space (current study) and between years (Høye et al., 2013). With temporal overlap with *Dryas* flowering causing particular scope for interplant competition for pollinators (see below), it is crucial to establish whether and how climate may affect the extent of such overlap.

During the last decade, elevational gradients have been increasingly used as a framework for studying environmental impacts on plant and pollinator communities (e.g., Adedoja, Kehinde, & Samways, 2018; Lara‐Romero, Seguí, Pérez‐Delgado, Nogales, & Traveset, 2019; Rafferty et al., 2020). Oftentimes changes in plant–pollinator interactions observed along elevational gradients appear to arise through changes in the respective species pools (Adedoja et al., 2018; Brittain, Kremen, & Klein, 2013; Lara‐Romero et al., 2019; Maglianesi, Blüthgen, Böhning‐Gaese, & Schleuning, 2015; Partida‐Lara et al., 2018; Simanonok & Burkle, 2014), with communities at higher elevations being characterized by less species and more generalized interactions (e.g., Hoiss et al., 2015; Ramos‐Jiliberto et al., 2010). In this study, we tried to minimize the effects of changes in plant diversity and composition (Simanonok & Burkle, 2014) by choosing all our study sites within a single, standardized vegetation type (*Dryas* heath). Unavoidably, flower abundances and plant diversity proved slightly lower with higher elevation (Figures S3 and S4), with a major change being the gradual disappearance of *C. tetragona* and *Vaccinium uliginosum* with increasing elevation. However, as neither species was particularly attractive to pollinators (see Figure 1), we are confident that variation in their abundances will have little if any effects on the patterns observed. Thus, the patterns detected seem more reflective of climatic impacts on the relative phenologies of a constant set of species than of climatic impacts on community composition as such.

In terms of the exact climatic cline occurring along our focal elevational gradient, we notice a surprising feature. Contrasting with general biogeographic patterns and with our own initial expectations, in our study system mean temperatures tend to *increase* with increasing elevation (0.4°C/100 m; Figure S2). This is due to two phenomena: a shading effect of the neighboring mountain on the lower part of our focal slope, and an inversion layer frequently forming in the valley and trapping warm air up slope. We found an imprint of the resulting climate on the extent of temporal overlap between species, with the flowering period of *P. radicatum* and *S. acaulis* sliding relatively earlier compared to *Dryas* at higher (and warmer) elevations (Figure 2a)—thereby increasing their niche overlap with the dominant species in a warmer climate (Figure 2b). Meanwhile, the overall community‐level niche overlap with *Dryas* decreased. How large and widespread such shifts in the relative timing and niche overlap of species may be is a question calling for urgent attention.

Climatic variation along the elevational gradient examined in 2016 (2°C; Figure S2) seemed large enough to shift *S. acaulis* flowering from being earlier than that of *Dryas* to coinciding with *Dryas* toward higher elevations (Figure 2a). Such shifts in space are matched by patterns earlier reported in time. Høye et al. (2013) showed that during the last two decades, the range of temperature variation at Zackenberg has been substantial, and large enough to change the relative flowering of *Dryas* and *S. acaulis*. In earlier (and warmer) years, the flowering of *S. acaulis* in “late” sites has become relatively earlier compared to *Dryas* flowering, whereas the opposite has been true for “early” sites. Thus, how the timing of the species will shift does depend on the warming experienced. For all we can tell, further warming is likely to shift the flowering of *S. acaulis* and *Dryas* closer together and increase their niche overlap (to the right in Figure 2a,b).

### 4.2 Arctic plants compete for pollinators

In this arctic system, some flowering plants seem much more attractive to insects than others: When in flower, *Dryas* attracts the main part of the flower visits, and saturates the pollen loads carried by insects. While accounting for only 31.2% of the flowers across sites, *Dryas* attracted more than 95% of flower visits and contributed 90.4% of the pollen grains carried by muscid flies. In fact, the pollen loads of muscid flies were dominated by *Dryas*, whenever there were at least some open *Dryas* flowers around.

The high attraction exerted by *Dryas* on pollinators seems to generate interspecific competition (instead of potential facilitative effects) for pollinators, since there is a limited number of pollinators to attract in the system. While a strict demonstration of pollen limitation should preferentially include experimental evidence that pollen subsidized by hand pollination result in increased seed set (Knight et al., 2005), the current results offer multiple parallel sources of evidence for the role of insect pollination and competition for pollination through the season.

First, flower visitation rate decreased with increasing flower densities. At high flower densities, visitation rates declined substantially (≥50 *Dryas* flowers on a plot resulted in less than 0.04 pollinators per *Dryas* flower, *n* = 177; Figure 3), implying that many flowers will receive no visitors at all.

Second, the pollen loads of the individual muscid flies examined were completely dominated by *Dryas* pollen (94.4% of pollinators; Figure 1c), and a large proportion (42%) of pollinators were actually devoid of other pollen. Thus, given a finite number of visits to each flower, the probability of being visited by a pollinator carrying conspecific pollen is likely to be low for plant species beyond *Dryas*, and further reduced with increasing density of flowering *Dryas*. Such interspecific effects were best quantified among *Dryas* and another abundant plant with substantial overlap in space and time: *S. acaulis*. The pattern found in pollen loads of *S. acaulis* on flies exhibits several features best compatible with interspecific competition for pollinators: at low densities of *Dryas*, increasing the density of flowering *S. acaulis* resulted in an increasing proportion of pollinators carrying *S. acaulis* pollen, but at high *Dryas* densities, no realistic *S. acaulis* density sufficed make more than a few flies out of a hundred carry the species’ pollen (Figure 4).

Most importantly, we believe that the current findings offer cues to the mechanisms behind the competitive effects. Temporarily high abundances of *Dryas* flowers appeared to cause monopolization of visitations and pollen loads carried by pollinators, and a subsequent reduction of seed set in flowers open during the flowering peak. As a gynodioecious plant, female individuals of *S. acaulis* rely on insects for pollen transfer. When *Dryas* was abundant, insect transport of *S. acaulis* pollen was no longer related to *S. acaulis* abundances per se (see Figure 4)—a pattern suggesting that *S. acaulis* flowers were ignored by the visitors because more attractive *Dryas* flowers were available. Thus, *S. acaulis* individuals that escaped the highest *Dryas* densities gained higher seed set. These observations of competition are akin to those of other systems with dominant flowering species, for example crops (Holzschuh et al., 2011) or invasive plants (Brown et al., 2002; Goodell & Parker, 2017). While highly attractive plants may also prop up local pollinator densities to the benefit of less attractive plants (Ghazoul, 2006; Losapio et al., 2019), the current findings show how the monopolization of pollen transport may strongly outweigh such benefits during the condensed flowering season of the Arctic.

### 4.3 Does competition for pollination explain seasonal patterns in seed set?

We note that the patterns in seed set here attributed to competition are also patterns in time, since high densities of *Dryas* are concentrated to a given period in the early summer (Figure 5a). Thus, other factors changing in time could potentially contribute to the patterns observed. Yet, we note considerations providing evidence for a competitive effect beyond temporal variation in plant performance.

In the current data, we found a general decline in seed set of *Dryas* with the advance of the summer, and this effect extended both to flowers visited by pollinators and to flowers from which pollinators were excluded (Figure 6). Seed set in *Dryas* was highest for flowers open before the flowering peak, when pollinators are active but competition by other flowers (including other *Dryas* flowers) is still limited (see Figure 5). This pattern might be an adaptation to the naturally short growing season of the Arctic, where investment in early seeds may be safer than seed set by late flowers, as the risk of failure increases with the proximity of the fall frost period (a risk demonstrated in an Alpine system by Kudo (1993). Meanwhile, despite the overall decline of seed set in late flowers, the fraction of seed set attributable to pollinator presence increased toward the late season (Figure 6). This pattern may potentially arise from plants investing more in producing outcrossed seeds (see, e.g., Free, 1966; Orford, Murray, Vaughan, & Memmott, 2016), thereby challenging the vagaries of the arctic fall. Meanwhile, seed set of *S. acaulis* showed an opposite temporal pattern with increasing seed set toward the end of the season, suggesting that the seasonal patterns in environmental conditions are not driving the patterns of seed set of plants in the area.

While we logically cannot exclude all seasonal imprints on the patterns observed, we propose that the patterns reported here reflect competition among plants for pollinators during the short flowering season of the High Arctic, and that seed set by other species is affected by their level of temporal overlap with *Dryas* as a particularly attractive flower resource. This proposal should clearly be validated by further experiments, including hand pollination of multiple species during different parts of the season. For now, it offers a strong hypothesis amenable to empirical testing.

### 4.4 Competition changes with a warmer climate

On top of the competition for pollinators caused by *Dryas*, we observed shifts in the relative timing and niche overlaps of flowering species. However, they are by no means the only climate‐driven changes observed in the system. Two changes in particular may have increased overall competition among plants for pollinators: First, in years of early phenology, the flowering season of plants is shorter and compressed toward the early season, that is there is shorter time for pollination (see Høye et al., 2013; Prevéy et al., 2019; Schmidt et al., 2016) and—given less compression of the timing of pollinator activity (Schmidt et al., 2016)—less pollinators per individual flower. This will increase the overall demand for pollen transport during this condensed flowering period, with no corresponding increase in pollinator availability. Second, changes in the pollinator community may be affecting the general availability of pollination services, and the competitive context of the plants. Interestingly, the pollen limitation during the peak flowering of *Dryas* may already have been accentuated by climate‐related effects: the abundance of the important muscid flies at our study area has decreased by 80% during the past 20 years (Loboda et al., 2017). Overall, the mismatch between flowering of plants and pollinator flight season at Zackenberg has apparently increased, as mainly caused by a shortening flowering period with no matching reduction in the insect activity period (Høye et al., 2013; Schmidt et al., 2016). The shortening of flowering season and increasing mismatch between pollinators could leave late‐emerging insects devoid of relevant resources to reproduce, thus contributing to changing of community composition (Gillespie et al., 2019) and to the decline of both muscid flies and other important pollinators (Høye et al., 2013; Loboda et al., 2017).

## 5 CONCLUSIONS

Progressing climate change may not only change the distribution of species, but also the distribution of their biotic interactions through changes in species phenologies. When species at the same trophic level compete for interaction partners, a shift in their abundances or relative timing may cause indirect effects through shifts in the strength of competition. In this paper, we reveal suggestive evidence for such a signature of climate change on biotic interactions in a rapidly warming Arctic: We find that during the short arctic summer, plants seem to compete for access to pollinators. The relative intensity of this competition is dictated by their phenological overlap, which is modified by climate. Recent climate change may already have aggravated these patterns—and will likely change competitive interactions further over the next few decades of rapid warming. Our findings suggest a worrying imprint of climate change, through a shift in the indirect interactions of arctic species. These findings point to shifts in the biotic interactions among arctic species, and urges a novel focus on shifts in horizontal interactions within trophic layers, as driven by ongoing climate change.

## AUTHOR CONTRIBUTION

M.T. and T.R. conceived the study, planned the field design, and wrote the first draft of the manuscript. M.T. performed the fieldwork in 2016. M.T., T.R., and T.K. performed the analyses. All the authors contributed substantially to revisions.

## Supporting information

Fig S1‐S4

## Data Availability

The complete data set is available at Dryad: https://doi.org/10.5061/dryad.nzs7h44pm.
