# Supplementary material for: Heated rivalries: Phenological variation modifies competition for pollinators among arctic plants
Source: Glob Chang Biol. 2020 Sep 11;26(11):6313–25. doi: 10.1111/gcb.15303 (PMC7693037; doi:10.1111/gcb.15303)
Supplement: Supplementary file 1 — Fig S1‐S4 [file GCB-26-6313-s001.docx]

Supporting Information

**Heated rivalries: phenological variation modifies competition for pollinators among arctic plants**

Mikko Tiusanen, Tuomas Kankaanpää, Niels Martin Schmidt & Tomas Roslin

Corresponding Author:

Mikko Tiusanen

mikko.tiusanen@helsinki.fi

This file includes:

**Supplementary Figure S1**

**Supplementary Figure S2**

**Supplementary Figure S3**

**Supplementary Figure S4**


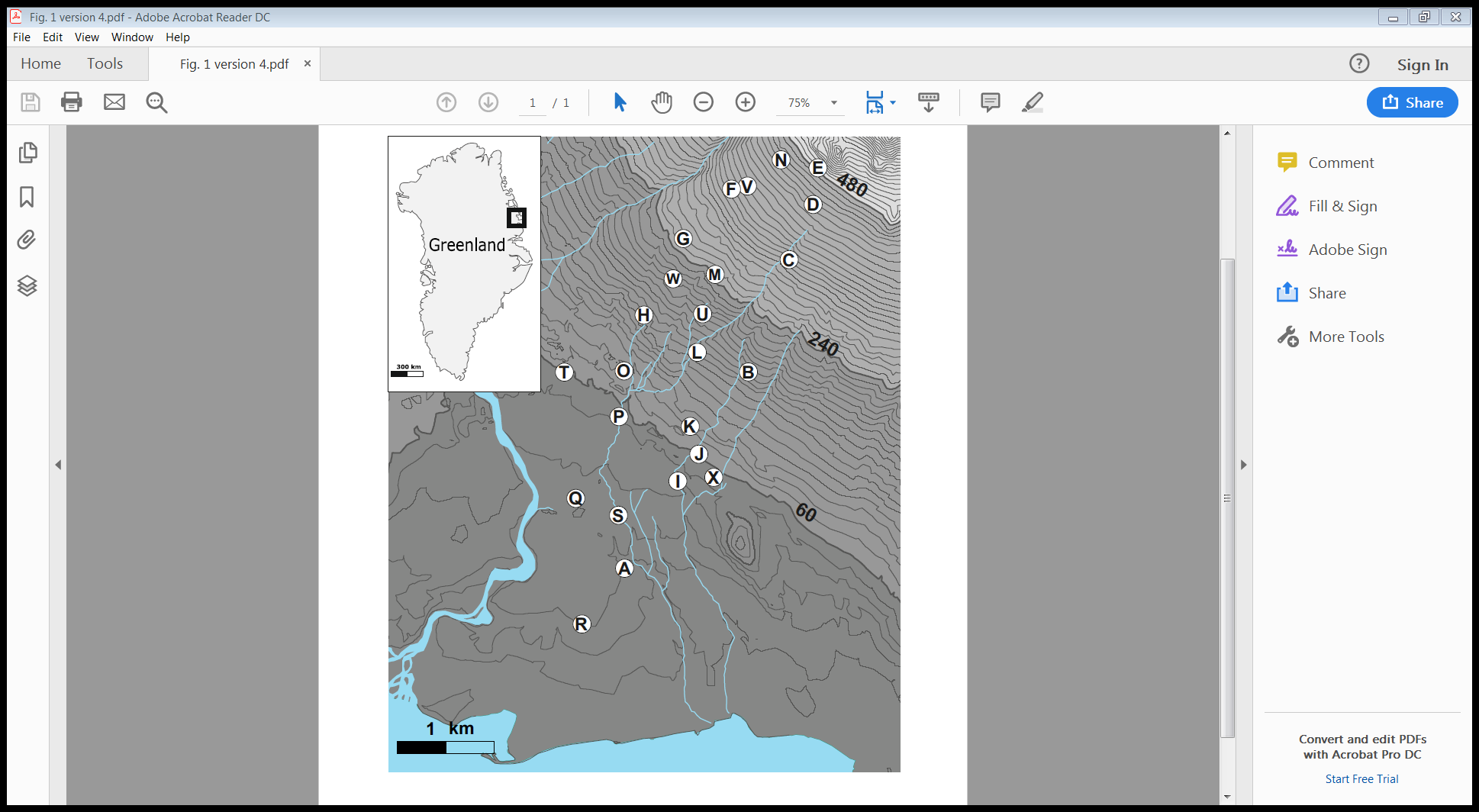
Supplementary Figure S1. Study sites within the Zackenberg valley (74° 30' N, 21° 00' W). Open circles (A–X) represent the 24 study sites. Gray lines represent elevational contours at 10 meter interval. Numbers on the picture mark the contours limiting the elevation zones (0-60m, 60-240m and 240-480m).

Supplementary Figure S2. Mean temperatures recorded along the elevational gradient in 2016. The error bars indicate the standard error of the mean, as based on six weekly mean temperatures from each site. The temperatures were recorded with air temperature and relative humidity loggers (EL-USB-2, Lascar electronics, U.K.) placed at a height of 10 cm and shielded from direct sun light by a white plastic dome. Note that the measurement points do not correspond directly to on our specific study sites, but to 19 plots along the very same mountain slope monitored by Tuomas Kankaanpää between May 22^nd^ and July 25^th^, 2016.


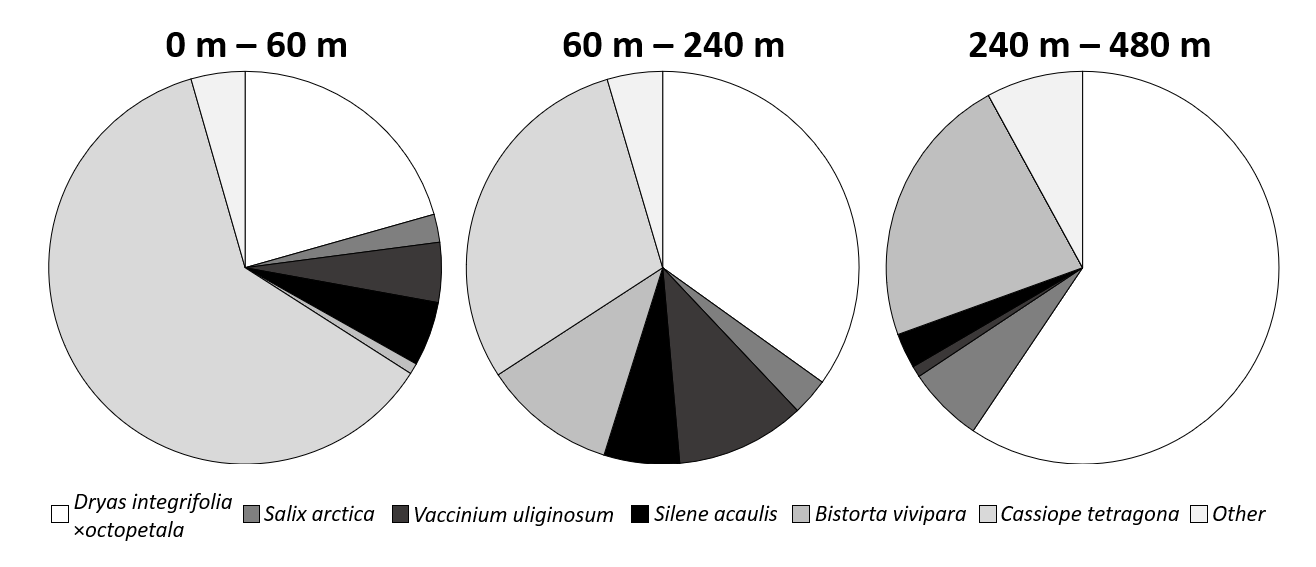


Supplementary Figure S3. Relative abundances of inflorescences of different plant species within the elevational zones identified in Fig. 1 (with 8 study sites within each zone). The pie charts from left to right represent data from 712 498; 383 148; and 218 314 individual flowers, respectively.

Supplementary Figure S4. The number of flowering species observed along the elevational gradient. Here, each data point represents an individual study site (n=24).
